# Grand Field Challenges for Cognitive Neuroergonomics in the Coming Decade

**DOI:** 10.3389/fnrgo.2021.643969

**Published:** 2021-03-08

**Authors:** Klaus Gramann, Ryan McKendrick, Carryl Baldwin, Raphaëlle N. Roy, Camille Jeunet, Ranjana K. Mehta, Giovanni Vecchiato

**Affiliations:** ^1^Biological Psychology and Neuroergonomics, Technische Universität Berlin, Berlin, Germany; ^2^Northrop Grumman Corporation, McLean, VA, United States; ^3^Department of Psychology, Wichita State University, Wichita, KS, United States; ^4^ISAE-SUPAERO, Université de Toulouse, Toulouse, France; ^5^Aquitaine Institute for Cognitive and Integrative Neuroscience, CNRS and University of Bordeaux, Bordeaux, France; ^6^Department of Industrial and Systems Engineering, Texas A&M University, College Station, TX, United States; ^7^Institute of Neuroscience, National Research Council of Italy, Parma, Italy

**Keywords:** cognitive neuroergonomics, mobile brain imaging, open access, virtual reality, embodied cognition, EEG, fNIRS

## Introduction

*Neuroergonomics* as defined by Raja Parasuraman is the study of “the brain at work and in everyday life” (Parasuraman, 2003). This rapidly growing research field aims at understanding human brain function underlying the many facets of human interaction with technical systems (Dehais et al., 2020). The term “cognition” is used to describe different processes (e.g., attention, memory, decision making) relevant to human-technology interaction. *Cognitive neuroergonomics*, then, can be defined as a section of neuroergonomics concerned with the investigation of the neural bases of those cognitive processes involved in the user's interaction with a technical system at work or during everyday life. One of the defining aspects of cognitive neuroergonomics is that it uses insights from analyzing neural dynamics in these settings to inform cognitive theory and models, as well as to improve our understanding of human brain function underlying cognition, in general.

To this end, new imaging methods are continuously adapted and used in a wide range of experimental scenarios that cover the entire area of ergonomics from highly controlled laboratory research protocols, to less controlled translational research, to research in the real world with little control over the factors of interest (Parada, 2018). This decreasing level of control is accompanied by an increasing level of ecological validity. Laboratory experiments provide very good control over experimental factors with high internal validity of the investigated constructs but often suffer from low levels of ecological validity. In contrast, real-world experiments might show low internal validity and lack of experimental control but provide high ecological validity that cannot be further improved. Here, the real world *is* the laboratory (Gramann et al., 2017). Furthermore, with increasing ecological validity, inter-*acting* with technical systems often involves expanding physical activity of the user. System interactions range from very low input (e.g., interaction with mobile devices; McKendrick, 2019) to larger scale interaction (e.g., Human-Robot Interaction, HRI; Tsarouchi et al., 2016) to very large scale interactions (e.g., assisted navigation; Wunderlich and Gramann, 2020). Active behavior is the basis for physically demanding workplaces as well as less physically challenging tasks that, nonetheless, require body, head and eye movements when users actively seek information or respond to external stimuli (Doshi and Trivedi, 2009). Traditionally, however, active behavior is not allowed in brain imaging protocols because established imaging modalities are usually too heavy to follow participants' movements and movement-related artifacts render the analyses of neural activity difficult (Makeig et al., 2009; Gramann et al., 2011).

With cognitive neuroergonomics maturing into a new research area with widespread research questions and methods, the focus should be put back into theory-driven studies of the human brain at work and in everyday life. Good scientific practices have to be adapted to allow for replicable science including the integration of new mobile imaging methods into the existing range of established imaging protocols. New findings have to be related to parameters known from established laboratory protocols and integrated into larger theoretical frameworks that allow for systematic replication as well as the development of robust parameters reflecting cognitive processes. From this perspective, it is our belief that the following challenges will have to be met to further develop this scientific field.

### Challenge 1: Bridging Basic, Translational, and Applied Research in Cognitive Neuroergonomics

**Figure 1:**
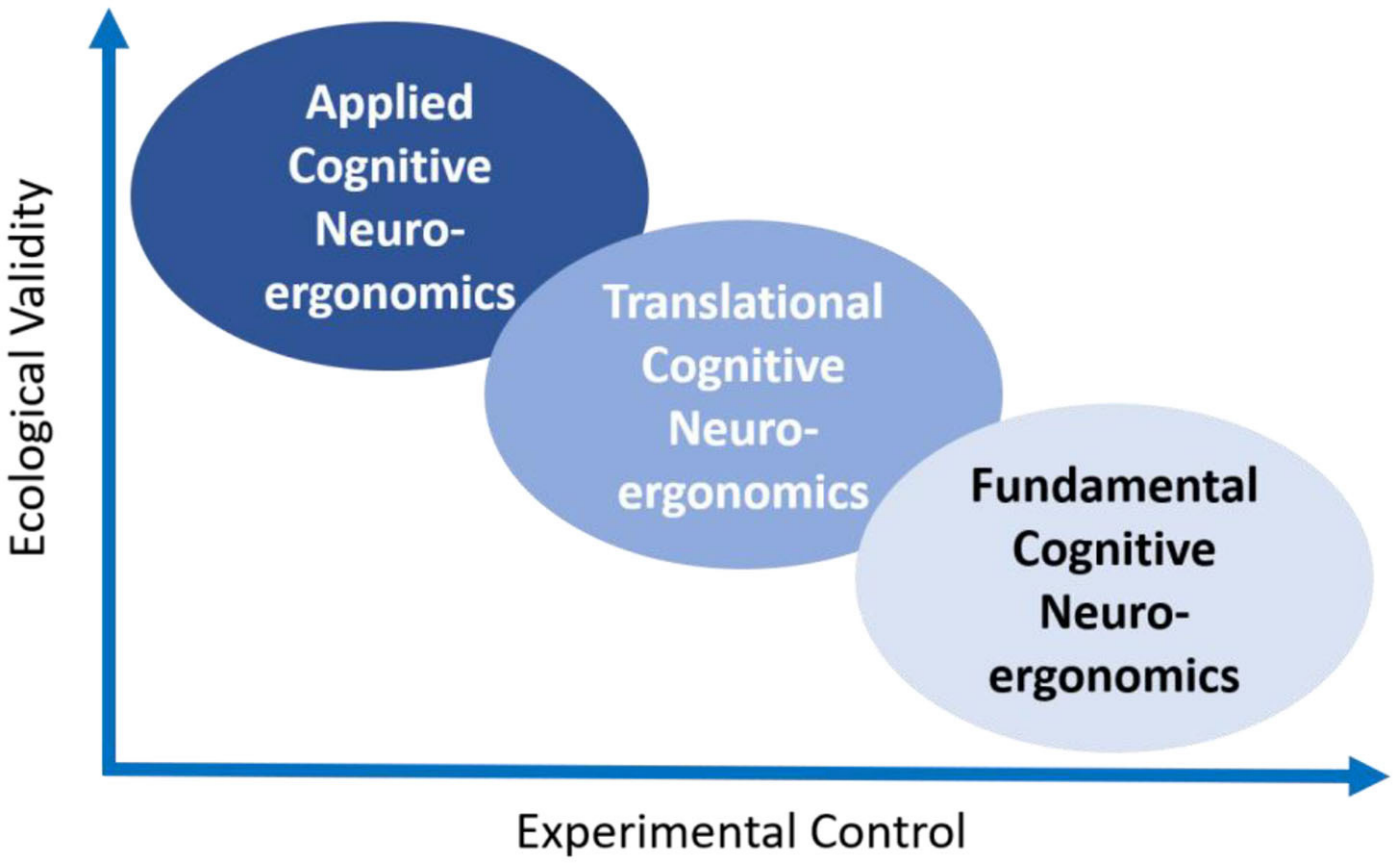
Experimental protocols in cognitive neuroergonomics regarding control and ecological validity.

Traditionally, research in cognitive neuroergonomics takes place in different environments spanning the entire space of protocols from fundamental to applied research (Figure 1). Fundamental research questions can be addressed in controlled laboratory settings and translated toward work environments (e.g., Gateau et al., 2018; Zhu et al., 2020a). In translational research, often expensive high-fidelity simulations are used to investigate more realistic cognitive and behavioral dynamics (Baldwin et al., 2017). These environments enable sufficient control over confounding factors while permitting repetitions of simulation scenarios to gain sufficient statistical power for analyzing a specific cognitive process of interest (Hollnagel, 2010). Finally, applied research takes place during interaction with technology in real-world settings which often do not allow for controlling how users interact with the system, providing limited or no control over external factors contributing to the behavior of users (Dehais et al., 2019).

Over the last decades, the majority of neuroergonomic studies took place in laboratories that provided the necessary infrastructure and housing for the heavy and susceptible imaging technologies. With the rise of lightweight mobile amplifiers, however, an increasing number of studies moved out of the lab using imaging methods in real-world settings (McKendrick et al., 2017; Chavarriaga et al., 2018; Protzak and Gramann, 2018).

All three areas of research in neuroergonomics are important and eventually converge to understand the cognitive and neural basis of human-technology interaction. To assure objectivity, reliability, and validity of the methods used, neuroergonomic studies have to allow for replication and falsifiability as two necessary pillars of a healthy scientific field that help to facilitate replication of key findings in the future.

Besides implementing good scientific practices, bridging fundamental, and applied studies in cognitive neuroergonomics can be achieved through the use of virtual reality (VR). VR allows for creating fully controlled and accurate virtual copies of real scenarios which are too expensive, dangerous or impossible to create in a standard experimental laboratory (Jeunet et al., 2018; e.g., Shi et al., 2020). VR can be useful to the design of technological interfaces leading to a reduction of research costs when compared to real scenarios (Chryssolouris et al., 2000). VR systems currently come with critical limitations including lower levels of fidelity when compared to real world scenarios (Hu et al., 2011), the lack of proper haptic feedback (Faure et al., 2020), and simulation sickness for a non-negligible percentage of the population (Duzmańska et al., 2018). But, VR technologies are rapidly improving and provide possible solutions to minimize many problems associated with low control, expensive access, and insufficient sample sizes in translational and applied neuroergonomics research in the next decades.

### Challenge 2: Imaging Methods for Embodied Cognitive Neuroergonomics

The majority of workplaces and interfaces require active behavior for successful interaction with a system. The programming and execution of motor action influences cognition as well as accompanying brain dynamics and it is thus imperative to understand cognitive processes during inter-*action* with a system (Gramann et al., 2011; Jungnickel and Gramann, 2016).

Mobile brain imaging approaches from mobile EEG (Gramann and Plank, 2019) and fNRIS (Izzetoglu et al., 2005) to Mobile Brain/Body Imaging (Jungnickel et al., 2019) allow for imaging human brain dynamics in actively behaving participants interacting with a technical system. Lightweight and mobile amplifiers exist for EEG and fNIRS that can be further combined and synchronized with additional physiological measures, motion capture, and other methods. Because of the active behavior of participants in mobile recordings, the acquired data is often contaminated with activity stemming from non-brain sources, usually considered artifact. Most mobile brain imaging studies thus require careful pre-processing of the recorded data (for EEG, see Klug and Gramann, 2020; for fNRIS, see Zhu et al., 2020b) and data-driven analyses to dissociate brain from non-brain activity (Makeig et al., 2009; Vitorio et al., 2017 for EEG and fNRIS, respectively). The increasing demands in preparation, technical challenges, and data analyses come with deeper insights into human brain dynamics only if the challenges of new mobile brain imaging methods can be overcome.

Even though improved mobile brain imaging methods allow for investigating naturalistic interactions with technical systems in the workplace and everyday settings (Gehrke et al., 2019; Wascher et al., 2020), these new methods likely come with potential changes in the extracted neural parameters (Gramann et al., 2018). Such differences have to be described and embedded in a systematic fashion to allow for understanding the theoretical and methodological framework and to foster convergence with results from standard laboratory protocols, leading directly to the third grand field challenge.

### Challenge 3: Generalizability of Physiological Parameters Reflecting Cognitive Processes

A variety of neurophysiological parameters have been identified in different experiments with diverging interpretations regarding their cognitive correlates. The advent of new imaging modalities and analysis approaches that allow for brain imaging in actively behaving participants, led to the discovery of new neural dynamics. It is important to embed new and diverging results in a theoretical framework rather than just accumulating new parameters that are disconnected from existing knowledge and established theory.

It is an eminent challenge in cognitive neuroergonomics to systematically compare physiological parameters across experimental tasks and neuroscientific methods to allow for identifying parameters that represent cognitive processes or user states independently of the tasks and methods used. In general, the eventual outcome of neurergonomic research, like any other research, should be a falsifiable model or theory that allows for predicting user behavior or user states based on neurophysiological data. If the model is successful in its predictions it is more likely to eventually provide generalizability (Baldwin and Penaranda, 2012). A systematic and concerted approach in the field of cognitive neuroergonomics should be able overcome some of these limitations. Advances in algorithms and data labeling have begun to show promise in these areas (McKendrick et al., 2019).

### Challenge 4: Open Access to Data and Protocols in Neuroergonomics

Reproducible scientific insights are essential to a democratic discourse and provide the basis for the design of a human-centered technology. Open Access (OA) approaches have been established over the last years to counteract restrictive paywalled scientific publication models that often come with high publication costs in established journals currently dominating science. While open access is an important and central aspect of barrier-free knowledge distribution, several additional aspects of publishing should be considered for a reproducible, transparent, and self-controlling scientific system.

For scientific practice to be reproducible and transparent, the experimental procedures, collected data and analyses approaches/code have to be published in combination with the manuscripts so that other researchers can replicate and test the results of a neuroergonomic study. Data sharing is beneficial to the neuroscience community in general (Sejnowski et al., 2014), but comes with its own hurdles including standardization of data formats, incentives to share data as well as its discoverability, among others (Wiener et al., 2016). Publication of data, protocols and code in open repositories (e.g., Github, OSF) might face opposition in some research areas due to, for example, financial interest or security restrictions in applied industrial research. Even where no such restrictions exist, such as in basic research pursuits, reproducibility and transparency practices are not yet the norm. To achieve these goals, it is fundamental to apply standardized approaches that have been developed in different scientific communities and for different modalities over the last years regarding data formatting (e.g., EEG-BIDS, Pernet et al., 2019) and reporting (e.g., CRED-NF for neurofeedback, Ros et al., 2020), alongside guidelines for data analyses and sharing (e.g., COBIDAS, Pernet et al., 2020). These formats and guidelines should be considered as future standards for neuroergonomics to allow for objective checks and balances in this rapidly growing scientific field. Finally, registered reports could be a future gold standard for reproducible basic research in cognitive neuroergonomics to allow for careful planning with sufficient sample sizes providing unbiased results and hopefully more valid neurophysiological parameters that reflect cognitive processes during interaction with technical systems.

## Conclusion

The rapidly growing scientific field of cognitive neuroergonomics benefits from new neuroscientific methods and technologies that allow bridging basic to applied research. Mobile imaging modalities in real world settings or near-realistic VR environments provide the opportunity to understand brain activity associated with natural interactions with technical systems. They also provide new opportunities to understand human brain functions associated with cognitive processes during active behavior in dynamically changing environments. These new technological opportunities will come with novel insights into the neural basis of cognitive states and processes. Reproducible scientific approaches in neuroergonomics based on open access and open protocols as well as open data will help to overcome some challenges of these new approaches allowing to address problems of replicability in the field (Barch and Yarkoni, 2013; Stanley et al., 2018). This way, cognitive neuroergonomics might provide new and applicable insights to improve human well-being when interacting with technical systems at work or during leisure.

## Author Contributions

All authors listed have made a substantial, direct and intellectual contribution to the work, and approved it for publication.

## Conflict of Interest

The authors declare that the research was conducted in the absence of any commercial or financial relationships that could be construed as a potential conflict of interest. The handling Editor declared a shared affiliation, though no other collaboration, with one of the authors RR.
